# KDM3A/Ets1 epigenetic axis contributes to PAX3/FOXO1‐driven and independent disease‐promoting gene expression in fusion‐positive Rhabdomyosarcoma

**DOI:** 10.1002/1878-0261.12769

**Published:** 2020-08-05

**Authors:** Lays M. Sobral, Hannah M. Hicks, Janet K. Parrish, Tyler S. McCann, Joseph Hsieh, Andrew Goodspeed, James C. Costello, Joshua C. Black, Paul Jedlicka

**Affiliations:** ^1^ Department of Pathology Anschutz Medical Campus University of Colorado Denver Aurora CO USA; ^2^ Cancer Biology Graduate Program Anschutz Medical Campus University of Colorado Denver Aurora CO USA; ^3^ Medical Scientist Training Program Anschutz Medical Campus University of Colorado Denver Aurora CO USA; ^4^ Department of Pharmacology Anschutz Medical Campus University of Colorado Denver Aurora CO USA; ^5^ Bioinformatics Shared Resource University of Colorado Cancer Center Aurora CO USA

**Keywords:** epigenetics, Ets1, Jumonji, KDM3A, metastasis, rhabdomyosarcoma

## Abstract

Rhabdomyosarcoma (RMS) is the most common soft tissue sarcoma in children and young adults. RMS exists as two major disease subtypes, oncofusion‐negative RMS (FN‐RMS) and oncofusion‐positive RMS (FP‐RMS). FP‐RMS is characterized by recurrent PAX3/7‐FOXO1 driver oncofusions and is a biologically and clinically aggressive disease. Recent studies have revealed FP‐RMS to have a strong epigenetic basis. Epigenetic mechanisms represent potential new therapeutic vulnerabilities in FP‐RMS, but their complex details remain to be defined. We previously identified a new disease‐promoting epigenetic axis in RMS, involving the chromatin factor KDM3A and the Ets1 transcription factor. In the present study, we define the KDM3A and Ets1 FP‐RMS transcriptomes and show that these interface with the recently characterized PAX3/FOXO1‐driven gene expression program. KDM3A and Ets1 positively control numerous known and candidate novel PAX3/FOXO1‐induced RMS‐promoting genes, including subsets under control of PAX3/FOXO1‐associated superenhancers (SE), such as MEST. Interestingly, KDM3A and Ets1 also positively control a number of known and candidate novel FP‐RMS‐promoting, but not PAX3/FOXO1‐dependent, genes. Epistatically, Ets1 is downstream of, and exerts disease‐promoting effects similar to, both KDM3A and PAX3/FOXO1. MEST also manifests disease‐promoting properties in FP‐RMS, and KDM3A and Ets1 each impacts activation of the PAX3/FOXO1‐associated MEST SE. Taken together, our studies show that the KDM3A/Ets1 epigenetic axis plays an important role in disease promotion in FP‐RMS, and provide insight into potential new ways to target aggressive phenotypes in this disease.

## Graphical abstract

Fusion‐positive rhabdomyosarcoma (FP‐RMS) is an aggressive pediatric cancer. The chromatin factor KDM3A and the downstream transcription factor Ets1 comprise a new epigenetic axis that works together with, as well as independently of, the driver PAX3/FOXO1 oncofusion to upregulate disease‐promoting gene expression and phenotypic properties in FP‐RMS. These findings offer new insights into potential ways to target aggressive properties in this disease.

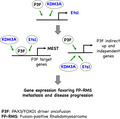

**Abbreviations**
shRNA: short hairpin ribonucleic acid
qRT‐PCR: quantitative reverse transcription–polymerase chain reaction
IVIS: *in vivo* imaging system
KD: knock down

## 1 Introduction

Rhabdomyosarcoma (RMS), the most common soft tissue cancer in the pediatric age group, is a malignancy of mesenchymal origin with skeletal muscle differentiation. Biologically and clinically, RMS predominantly exists as two distinct disease subtypes [1, 2, 3]. Fusion‐negative RMS (FN‐RMS) usually affects a younger age group, arises in more central anatomic sites, typically shows ‘embryonal’ (‘ERMS’) histology, and is associated with better outcomes (> 70% 5‐year survival with appropriate chemotherapy). Fusion‐positive RMS (FP‐RMS), on the other hand, usually occurs in older children, arises in more peripheral anatomic sites, typically shows ‘alveolar’ (‘ARMS’) histology, and is associated with much less favorable outcomes. Definitionally a high‐risk disease, FP‐RMS is less chemoresponsive, more metastatic, and more prone to recurrence than FN‐RMS. Overall 5‐year survival for FP‐RMS is < 50% and falls below 20% for patients with metastatic or recurrent disease [1, 4].

On a molecular level, FN‐RMS is a heterogeneous disease, frequently associated with mutations in receptor tyrosine kinase signaling axes, and, less commonly, other known oncogenic pathways [1, 2, 5]. FP‐RMS, in contrast, is a prototypical, mutationally quiescent, cancer of childhood, driven by fusion oncogenes arising from chromosomal translocations [1, 2, 3, 5]. These translocations fuse the amino terminus of the PAX3 or PAX7 gene to the carboxy terminus of the FOXO1 gene. PAX3 and PAX7, both transcription factors involved in normal myogenesis, supply DNA‐binding domains. FOXO1, also a transcription factor, provides a transcriptional activation domain. PAX3/FOXO1 is the driver oncofusion in 70% of FP‐RMS and promotes phenotypes critical to both sarcoma growth and dissemination. The less common PAX7/FOXO1 oncofusion has been less studied. Interestingly, while fusion subtype does not affect survival of patients presenting without clinically overt distal metastases, patients presenting with PAX3/FOXO1 metastatic disease appear to have worse outcomes than those with PAX7/FOXO1 metastatic disease [6]. Thus, PAX3/FOXO1 FP‐RMS is a particularly aggressive disease.

Mechanistically, PAX3/FOXO1 is an aberrant regulator of gene expression, whose transcriptomic effects include induction of genes promoting cell proliferation, survival, motility, and invasion, as well as altered expression of myogenic program genes resulting in impaired differentiation [1, 2, 3, 4]. Recent studies have uncovered critical roles for epigenetic mechanisms in the disease‐driving effects of PAX3/FOXO1 and the pathogenesis of FP‐RMS. This includes activation of enhancers and so‐called ‘superenhancers (SEs)’ by PAX3/FOXO1 itself [7]; utilization of the chromatin factors JARID2, EZH2, CHD4, and BRD4 [7, 8, 9, 10]; and utilization of myogenic transcription factor networks [7]. With the PAX3/FOXO1 oncofusion itself a very difficult therapeutic target, epigenetic mechanisms critically contributing to PAX3/FOXO1 action present exciting alternative targeting opportunities, as recently illustrated for BRD4 [7]. However, capitalizing on these opportunities depends on fully unraveling the epigenetic code underpinning PAX3/FOXO1‐driven oncogenesis.

Our recent studies identified the epigenetic regulator KDM3A (JMJD1A/JHDM2A), a member of the Jumonji‐domain histone demethylase family [11], as a novel and potent disease‐promoting factor in both FN‐RMS and FP‐RMS [12]. The same studies also identified the Ets1 transcription factor as a downstream mediator contributing to KDM3A effects. In the present study, we sought to further understand how this novel KDM3A/Ets1 epigenetic regulatory axis contributes to PAX3/FOXO1‐driven gene expression and FP‐RMS pathogenesis.

## 2 Materials and methods

### 2.1 Cell lines

The patient‐derived RMS cell lines RD, SMS‐CTR, Rh30, and Rh41, and culture conditions, have been described [12]. Rh30 and Rh41 FP‐RMS cells engineered to express doxycycline‐inducible nontargeting scrambled control short hairpin ribonucleic acid (shRNA) or PAX3‐FOXO1 fusion oncoprotein‐targeting shRNA were kindly provided by Mark Hatley, St. Jude Children's Research Hospital, and were cultured as described [13]. All cell lines were authenticated at our institution by short tandem repeat profiling and repeatedly verified to be mycoplasma‐free.

### 2.2 Stable depletion of gene expression

Stable, shRNA‐mediated, depletion of KDM3A, Ets1, and MEST expression in RMS cells was performed as previously described, using lentiviral delivery [12]. Scrambled (nontargeting control) shRNA (Addgene plasmid 1864), and KDM3A and Ets1 targeting shRNAs are described in Ref. [14] and have been previously validated in RMS cell lines used here [12]; shRNAs 1 and 2 for MEST correspond to TRCN0000075318 and TRCN0000075320 (Sigma Mission shRNAs, distributed via the University of Colorado Cancer Center Functional Genomics Core Facility). Following transduction, cells were selected with 1 µg·mL^−1^ of puromycin for 3–4 days, and depletion of gene expression was verified using protein immunoblotting, or quantitative reverse transcription–polymerase chain reaction (qRT‐PCR) (for MEST, for which reliable antibodies were not identified). For PAX3/FOXO1 depletion experiments, control and targeting shRNAs were induced by treatment of cells with 100 ng·mL^−1^ doxycycline for 5 days.

### 2.3 Protein immunoblotting

Protein immunoblotting was performed as previously described [12]. Primary antibodies used were as follows: Ets1 (Cell Signaling, Danvers, MA, USA; #14069; 1 : 1000); FOXO1 (Cell Signaling Technologies; #2880; 1 : 1000); and tubulin (Sigma, St. Louis, MO, USA; T5168; 1 : 20 000).

### 2.4 Quantification of RNA expression

Cells were harvested at 70–80% confluence in TRIzol (Invitrogen, Waltham, MA, USA), and RNA was extracted per manufacturer instructions. RNA levels of specific transcripts were assessed by qRT‐PCR (using qScript Super Mix and Perfecta SYBR Green Fast Mix; Quantabio) with RPL19 RNA as the internal control (primers are listed in Table S1).

### 2.5 Growth and invasion assays

Clonogenic growth assays and transendothelial invasion assays in RD, SMS‐CTR, Rh30, and Rh41 RMS cells were performed as previously described [12].

### 2.6 *In vivo* xenograft studies

Tail vein injection xenograft model studies were performed as previously described [12]. Briefly, 1 × 10^6^ Scramble control or shEts1 Rh30 cells, each additionally expressing a luciferase reporter (described in Ref. [14]), were injected into the tail vein of NOD‐SCID/Gamma mice (9–10 animals/group). Metastasis development was monitored weekly using *in vivo* imaging system (IVIS) imaging following administration of luciferin. All animal experiments were in compliance with ethical regulations as approved by our Institutional Animal Care and Use Committee.

### 2.7 Transcriptome analysis

Transcriptome profiling was performed on triplicate samples of FP‐RMS Rh30 and Rh41 cells, transduced with scrambled control shRNA, KDM3A‐sh1, KDM3A‐sh2, Ets1‐sh1, or Ets1‐sh2 (described in Ref. [14]), and all previously validated in RMS cell lines used here [12]). RNA was isolated using TRIzol (Invitrogen) and further purified using the Qiagen (Germantown, MD, USA) MinElute column kit. Following verification of KDM3A and Ets1 depletion and downregulation of the downstream gene MCAM [12], samples were submitted to Novogene Corporation Inc (Sacramento, CA, USA) for analysis of RNA quality, library preparation, and paired‐end (PE150) mRNA next‐generation sequencing on an Illumina platform. To determine gene expression values, raw fastq files were processed with rsem [15] (v1.3.1) using the bowtie2 [16] (v2.3.4.1) sequence aligner with default parameters. Reads were mapped to the Ensembl v92 transcriptome (hg38). Differential gene expression was calculated using the voom function in limma [17], comparing expression in KDM3A‐sh1 and KDM3A‐sh2 samples to shControl samples, and Ets1‐sh1 and Ets1‐sh2 samples to shControl samples; low abundance genes were removed (total count across all samples < 200). Transcriptome data are shown in Table S2. Gene Set Enrichment Analysis (GSEA) was performed using gsea software [18], with KDM3A and Ets1 transcriptomes as the rank‐ordered datasets. Gene sets with *P* < 0.05 (after 1000 gene set permutations) were deemed to be enriched in each group. Transcriptome overlap (Venn) analysis used genes differentially expressed at *P* < 0.05 and log_2_FC < −0.2 (for genes down with KDM3A/Ets1 knockdown (KD)] or log_2_FC > 0.2 [for genes up with KDM3A/Ets1 KD), and the online tools www.interactivenn.net or genevenn.sourceforge.net; overlap analysis with PAX3/FOXO1‐regulated genes used data from [7]. Gene Ontology (GO) analysis was performed using the National Institutes of Health Database for Annotation, Visualization, and Integrated Discovery (DAVID) public online tool (http://david.abcc.ncifcrf.gov/) using Biological Process GO terms.

### 2.8 Chromatin immunoprecipitation

Cells were cross‐linked with 1% formaldehyde, followed by quenching with 0.125 m glycine, both at room temperature. Cells were washed 2× with ice‐cold PBS, collected in ice‐cold PBS by scraping, pelleted, and resuspended in cell lysis buffer (5 mm PIPES, pH 8.0; 85 mm KCl; 0.5% NP‐40). Following incubation on ice for 10 min, a nuclear‐enriched fraction was collected by centrifugation for 5 min at 2350 ***g*** at 4 °C. The pellet was resuspended in ChIP lysis buffer (50 mm Tris/HCl, pH 8.1; 10 mm EDTA; 1% SDS; 0.1 mm PMSF; 1 µg·mL^−1^ each of aprotinin and leupeptin) on ice and subjected to sonication in the Bioruptor Pico apparatus (Diagenode, Denville, NJ, USA) for 20 cycles (each 30 s on/ 30 s off) at high power. The resulting sonicate was centrifuged at 21 130 ***g*** for 10 min at 4 °C to pellet debris. The supernatant was collected, and chromatin was quantified and stored in 10–20 µg aliquots at −80 °C. Following verification of appropriate chromatin fragmentation, 10 µg of chromatin was diluted in 500 µL of ChIP dilution buffer (16.7 mm Tris/HCl, pH 8.1; 167 mm NaCl; 1.2 mm EDTA; 0.2% SDS; 0.84% Triton X‐100) and precleared by addition of 50 µL of protein A/G agarose beads (Thermo Scientific, Waltham, MA, USA, #20423) and rotation for 1 h at 4 °C. Samples were spun briefly to pellet the beads. Fifty microlitre (10%) of supernatant was set aside as Input. For ChIP, antibody (H3K27Ac; Active Motif, Carlsbad, CA, USA, #39135; H3; Abcam, Cambridge, MA, USA, #1791) was added to 500 µL of the remaining precleared chromatin preparation, and the samples were incubated overnight with rotation at 4 °C. 20 µL of magnetic protein A/G beads (EMD Millipore, Burlington, MA, USA, #16‐663) was added, and the samples were rotated at 4 °C for 4 h. The ChIP‐bead complexes were sequentially washed: 2× with low salt buffer (20 mm Tris/HCl pH 8.1, 150 mm NaCl, 2 mm EDTA, 0.1% SDS, 1% Triton X‐100); 2× with high salt buffer (20 mm Tris/HCl pH 8.1, 500 mm NaCl, 2 mm EDTA, 0.1% SDS, 1% Triton X‐100); 2× with LiCl buffer (10 mm Tris pH 8.1, 1 mm EDTA, 0.5 m LiCl, 1% NP‐40, 1% deoxycholic acid); and 2x with TE buffer (10 mm Tris pH 8.1, 1 mm EDTA). Cross‐links were reversed and ChIP DNA was recovered by: addition of 200 µL of Elution buffer (0.1 m NaHCO_3_, 1% SDS) and 0.2 m NaCl, followed by overnight incubation at 65 °C; addition of 20 µg·mL^−1^ RNase A and incubation at 37 °C with for 1 h; addition of 100 µg·mL^−1^ proteinase K and incubation at 55 °C for 1 h; and phenol/chloroform extraction and ethanol precipitation. Dry ChIP DNA was resuspended in 50 µL of H_2_O and analyzed for enrichment of specific genomic regions, relative to input DNA, by quantitative polymerase chain reaction (primer sequences are listed in Table S1).

## 3 Results

### 3.1 Ets1 is a disease‐promoting factor in RMS, with particularly potent phenotypic effects in FP‐RMS

**Fig. 1:**
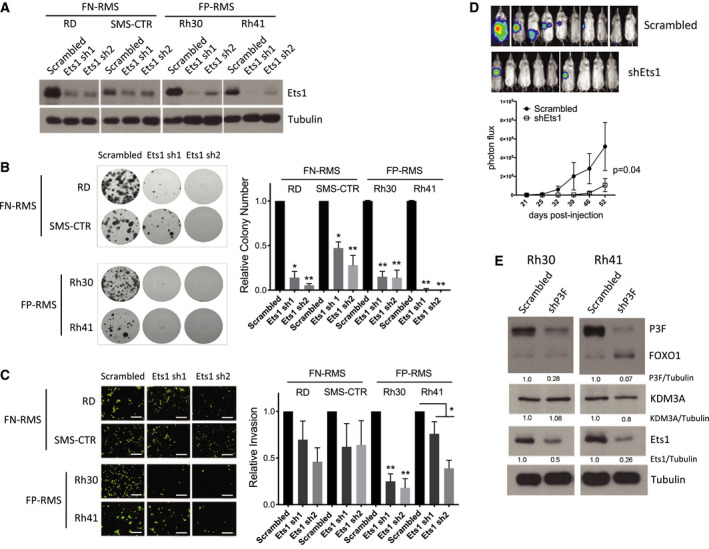
Ets1 exerts disease‐promoting effects in RMS and is under positive regulatory control of P3F in FP‐RMS. (A) Stable, shRNA‐mediated, depletion of Ets1 protein expression in RMS cells, as determined by protein immunoblotting with tubulin as loading control (*n* = 3, representative data shown). (B) Effects of Ets1 shRNA‐mediated depletion on clonogenic growth of RMS cells. Shown are representative images of colonies formed, and quantifications of colony count data. The latter are plotted as mean and standard error of the mean of three independent experiments (*n* = 3), each performed in triplicate, with the control set to 1; *P*‐values were determined using one‐way analysis of variance (1‐way ANOVA) with multiple comparisons; *P < 0.05, **P < 0.01. (C) Effects of Ets1 depletion on transendothelial invasion by RMS cells. Shown are representative images (scale bar = 50 µm), and quantifications, of invaded cells. Data show mean and standard error of the mean of three independent experiments (*n* = 3), each performed in duplicate, with the control set to 1; *P*‐values were determined using one‐way ANOVA with multiple comparisons, or two‐way Student's *t*‐test with unequal variance (Rh41 data); *P < 0.05, **P < 0.01. (D) Effects of Ets1 depletion on metastasis in the tail vein injection model. Scrambled control or shEts1 Rh30 FP‐RMS cells, each additionally expressing a luciferase reporter, were injected into the tail vein of NOD‐SCID/gamma mice [*n* = 10 (scrambled) and 9 (shEts1)]. Metastasis development was monitored using IVIS imaging following administration of luciferin. Top panel shows IVIS imaging data at end of experiment. Bottom panel shows IVIS quantification data (mean and standard error of the mean of photon flux; *P*‐value from 2‐way ANOVA with repeated measures). (E) Effects of PAX3/FOXO1 (P3F) depletion on KDM3A and Ets1 protein levels in FP‐RMS cells. Representative immunoblots and mean of densitometric quantifications from two independent experiments (*n* = 2), normalized to scrambled shRNA control (FOXO1 antibody was used for detection of P3F, as done previously [13]).

Our previous studies identified the epigenetic regulator KDM3A as a potent new disease‐promoting factor in both FN‐RMS and FP‐RMS [12]. Furthermore, similar to our prior studies in Ewing's sarcoma (ES) [14], we identified the Ets1 transcription factor as an important downstream contributor to KDM3A regulation of the disease‐promoting gene MCAM [12]. The same studies revealed Ets1 to be highly expressed in RMS relative to ES [12], raising the question whether Ets1 itself plays an important disease‐promoting role in this disease. We therefore examined the phenotypic effects of Ets1 shRNA‐mediated depletion in patient‐derived FN‐RMS (RD and SMS‐CTR) and FP‐RMS (Rh30 and Rh41) cell lines. Similar to our prior studies [12], we employed the clonogenic assay and the transendothelial invasion assay to evaluate properties important for sarcoma growth and dissemination, respectively. In all four cell lines, depletion of Ets1 resulted in potent inhibition of clonogenic growth (Fig. 1A,B). Ets1 depletion in FN‐RMS RD and SMS‐CTR cells resulted in a trend toward diminished transendothelial invasive ability, but the effects, over multiple experiments, were variable and did not reach statistical significance (Fig. 1C). In contrast, in FP‐RMS Rh30 cells, Ets1 depletion resulted in potent and consistent reduction in transendothelial invasion (Fig. 1C). In FP‐RMS Rh41 cells, Ets1 depletion resulted in diminished transendothelial invasive ability that was not as potent as in Rh30 cells, but was also consistent and statistically significant over multiple experiments (Fig. 1C). To determine whether the potent growth and invasion‐promoting effects of Ets1 in FP‐RMS Rh30 cells translate into increased metastasis *in vivo*, we turned to the tail vein experimental metastasis assay. Ets1 depletion in FP‐RMS Rh30 cells resulted in significantly reduced metastatic burden in this assay (Fig. 1D). Thus, Ets1 exerts growth‐promoting effects in RMS and promotes transendothelial invasion and metastasis in FP‐RMS.

### 3.2 Ets1 expression is under positive regulatory control of PAX3/FOXO1 in FP‐RMS

The phenotypic effects of Ets1 were quite similar to those of KDM3A, which we have previously shown to positively control the expression of Ets1 in RMS [12]. The phenotypic effects of both KDM3A and Ets1 were also similar to the known growth and invasion‐promoting effects of the PAX3/FOXO1 driver oncofusion in FP‐RMS [13]. To determine whether KDM3A, Ets1, or both might be downstream of PAX3/FOXO1, we examined the effects of PAX3/FOXO1 shRNA‐mediated depletion on their expression in FP‐RMS cells. PAX3/FOXO1 depletion resulted in robust diminution of Ets1 expression levels in both Rh30 and Rh41 FP‐RMS cells, but little to no change in KDM3A expression levels (Fig. 1E). Thus, Ets1 expression is under positive regulatory control of PAX3/FOXO1, in addition to KDM3A, in FP‐RMS. Increased expression of KDM3A in FP‐RMS [12], on the other hand, appears to be mediated by mechanisms other than the PAX3/FOXO1 oncoprotein.

### 3.3 KDM3A and Ets1 each exert broad regulatory control over the FP‐RMS disease‐promoting transcriptome

**Fig. 2:**
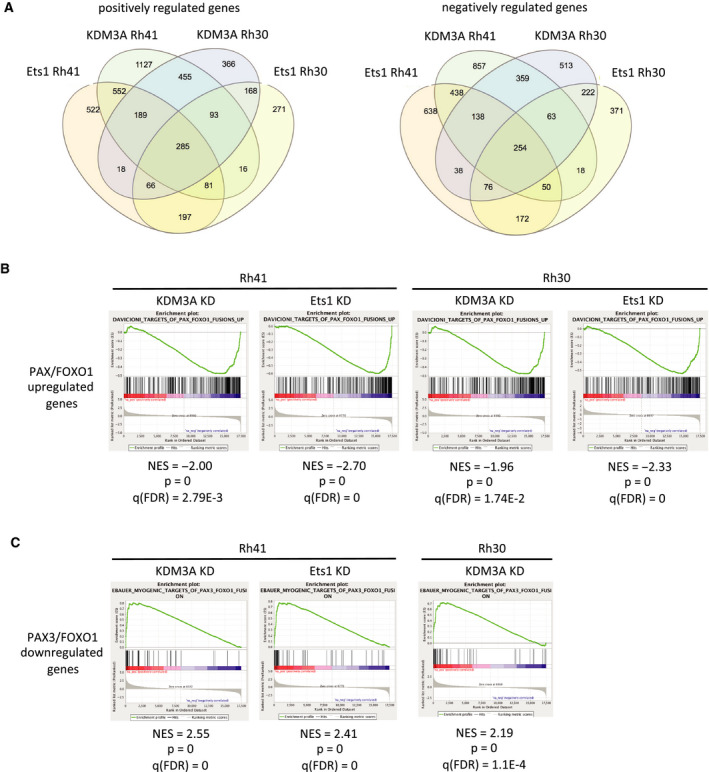
KDM3A and Ets1‐regulated transcriptomes and their relationships to P3F‐controlled gene expression in FP‐RMS. (A) Overlap (Venn) analysis of KDM3A and Ets1‐regulated transcriptomes in Rh30 and Rh41 FP‐RMS cells (positively and negatively regulated genes are inferred from genes down and up, respectively, upon KDM3A and Ets1 KD (*n* = 3 for each, including two independent shRNAs each for KDM3A and Ets1; see Materials and methods). (B, C) GSEA showing relationships of KDM3A and Ets1 transcriptomes to P3F upregulated (B) and downregulated (C) genes (NES: Normalized Enrichment Score; FDR: False Discovery Rate; see also Table 1).

Given the phenotypic concordance among KDM3A, Ets1, and PAX3/FOXO1, and the epistatic relationships of PAX3/FOXO1 and KDM3A relative to Ets1, we next sought to understand how KDM3A and Ets1 contribute to the regulatory control of the disease‐promoting transcriptome in FP‐RMS. To define the KDM3A and Ets1 regulated transcriptomes in FP‐RMS, we performed RNAseq analysis of control (scrambled shRNA), KDM3A KD, and Ets1 KD Rh41 and Rh30 cells. The transcriptome data are summarized in Figs 2A and S1. Consistent with the known functions of KDM3A and Ets1 as activators of gene expression [11, 19], and with Ets1 downstream of KDM3A [12], approximately one third of genes positively controlled by KDM3A were also positively regulated by Ets1, while roughly one half of genes positively controlled by Ets1 were also positively regulated by KDM3A, in each cell line (Figs 2A and S1). The KDM3A and Ets1 regulated transcriptomes showed substantial conservation between the two different FP‐RMS cell lines, with approximately one third of all genes positively controlled by each factor in Rh41 cells also similarly regulated in Rh30 cells, and, conversely, nearly two thirds of KDM3A positively controlled genes and one half of Ets1 positively controlled genes in Rh30 cells showing similar regulation in Rh41 cells (Figs 2A and S1). As expected, our stable depletion studies also revealed KDM3A and Ets1 downregulated genes (Figs 2A and S1), possibly representing indirect, or direct repressive, mechanisms of regulation.

GSEA of the transcriptome data revealed that, consistent with their phenotypic effects, KDM3A and Ets1 each positively control gene expression programs related to cell proliferation, cell motility, and metastasis, in both Rh30 and Rh41 cells (Table 1). Moreover, KDM3A and Ets1 also positively control a group of genes commonly overexpressed in pediatric cancers relative to normal tissues (‘Whiteford Pediatric Cancer Markers’ [20]; Table 1). Strikingly, GSEA further revealed strong overlaps of the KDM3A and Ets1 transcriptomes with the PAX3/FOXO1‐regulated transcriptome in FP‐RMS. Specifically, KDM3A and Ets1 KD each resulted in downregulation of PAX/FOXO1‐activated genes, while KDM3A KD in both cell lines, and Ets1 KD in Rh41 cells, resulted in upregulation of PAX3/FOXO1‐repressed genes, including myogenic differentiation genes (Table 1 and Fig. 2B,C). Thus, KDM3A and Ets1 each positively control disease‐promoting gene expression programs in FP‐RMS, and their gene expression regulatory effects strongly mirror those of the PAX3/FOXO1 driver oncofusion.

**Table 1:** GSEA data for KDM3A and Ets1 transcriptomes. Note that the ‘Ebauer Targets of PAX3/FOXO1 Fusion Up’ gene set corresponds to genes up with PAX3/FOXO1 KD, and thus represents PAX3/FOXO1‐repressed genes [53]).

GSEA gene set	KDM3A KD						Ets1 KD					
	Rh41			Rh30			Rh41			Rh30		
	NES	*P*‐val	FDR *q*‐val	NES	*P*‐val	FDR *q*‐val	NES	*P*‐val	FDR *q*‐val	NES	*P*‐val	FDR *q*‐val
Growth												
CHANG_CYCLING_GENES	−2.73	0	0	−1.49	0	1.28 × 10^−1^	−2.41	0	0			
ZHANG_PROLIFERATING_VS_QUIESCENT	−1.89	0	9.68 × 10^−3^				−2.53	0	0	−2.14	0	7.56 × 10^−4^
Metastasis												
WU_CELL_MIGRATION				−1.59	0	8.84 × 10^−2^	−2.00	0	4.48 × 10^−3^	−2.00	0	5.99 × 10^−3^
SARRIO_EPITHELIAL_MESENCHYMAL_TRANSITION_UP	−2.45	0	0	−1.86	0	2.88 × 10^−2^	−2.39	0	0	−1.56	0	8.72 × 10^−2^
WINNEPENNINCKX_MELANOMA_METASTASIS_UP	−2.59	0	0	−1.88	0	2.73 × 10^−2^	−2.40	0	0			
JECHLINGER_EPITHELIAL_TO_MESENCHYMAL_TRANSITION_UP							−1.57	6.69 × 10^−3^	7.20 × 10^−2^	−1.89	0	1.56 × 10^−2^
Other												
WHITEFORD_PEDIATRIC_CANCER_MARKERS	−2.71	0	0	−2.12	0	4.67 × 10^−3^	−2.42	0	0	−1.45	1.45 × 10^−2^	1.43 × 10^−1^
FP‐RMS; PAX3/FOXO1 activated												
DAVICIONI_TARGETS_OF_PAX_FOXO1_FUSIONS_UP	−2.00	0	2.79 × 10^−3^	−1.96	0	1.74 × 10^−2^	−2.70	0	0	−2.33	0	0
FP‐RMS; PAX3/FOXO1 repressed												
EBAUER_TARGETS_OF_PAX3_FOXO1_FUSION_UP	2.45	0	0	1.96	0	7.77 × 10^−3^	2.14	0	1.65 × 10^−4^			
EBAUER_MYOGENIC_TARGETS_OF_PAX3_FOXO1_FUSION	2.55	0	0	2.19	0	1.10 × 10^−4^	2.41	0	0			

### 3.4 KDM3A and Ets1‐regulated transcriptomes intersect with the PAX3/FOXO1 cistrome and transcriptome

**Fig. 3:**
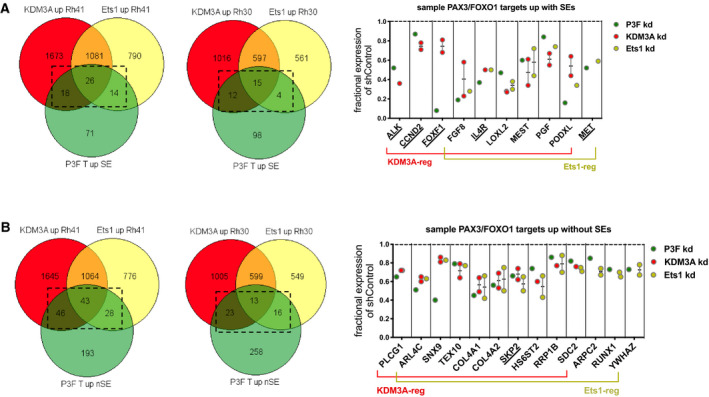
KDM3A and Ets1 contribute to regulatory control of P3F target genes. (A) Overlap (Venn) analysis of KDM3A and Ets1 ‘up’ transcriptomes in Rh30 and Rh41 cells (as inferred from genes down in expression upon KDM3A or Ets1 KD), and PAX3/FOXO1‐activated target genes associated with SEs, as defined by Gryder *et al*.[7]. Left: Venn diagram data (‘P3F T up SE’: PAX3/FOXO1 Targets up with SEs; dashed box: gene overlaps of interest). Right: selected known (underlined) and novel candidate (all others) disease‐promoting genes in FP‐RMS. For KDM3A and Ets1, each data point represents mean fractional expression (KDM3A‐sh1 and KDM3Ash‐2, or Ets1‐sh1 and Ets1‐sh2, relative to shControl, each *n* = 3) in one cell line; when a gene was identified in the regulated transcriptome in both cell lines, these data are shown as separate points (one for each cell line, along with overall mean and standard error of the mean); data from effects of PAX3/FOXO1 KD on gene expression are from Gryder *et al*. [7]. (B) Same overlap (Venn) analysis as ‘A’, but focused on PAX3/FOXO1‐activated target genes not associated with SEs (‘P3F T up nSE’), as defined by Gryder *et al*.[7] (dashed box: gene overlaps of interest).

A recent study performed an in‐depth integrative genomic analysis to identify genes under regulatory control of the PAX3/FOXO1 driver oncofusion in FP‐RMS [7]. This analysis defined 439 genes as high‐confidence PAX3/FOXO1 direct targets under positive regulatory control of the oncoprotein. Additional characterization of the genomic context and cistrome associated with these PAX3/FOXO1‐activated target genes showed 129 (29%) of these to be associated with SEs [7]. Gene overlap analysis with our transcriptome data revealed that KDM3A and Ets1 contribute to positive regulatory control of PAX3/FOXO1 directly activated and SE‐associated genes in FP‐RMS cells (Fig. 3A). Notably, this group of genes directly bound by PAX3/FOXO1, associated with SEs, and coregulated by KDM3A or/and Ets1, includes the following: known disease‐promoting genes in FP‐RMS (ALK, CCND2, FOXF1, IL‐4R, LOXL2, and MET) [1, 21, 22, 23, 24, 25]; other cancer‐promoting genes not previously studied in RMS (FGF8, PGF, and PODXL) [26, 27, 28, 29]; and MEST, a gene of poorly understood function upregulated in expression in RMS [30] (Fig. 3A). For the remaining 310 genes directly bound and activated by PAX3/FOXO1, but not associated with SEs [7], gene overlap analysis revealed that KDM3A and Ets1 also contribute to positive regulatory control of these genes in FP‐RMS (Fig. 3B). Similar to the SE‐associated genes, this coregulated gene group includes a known FP‐RMS disease‐promoting gene, SKP2 [31], and 13 additional genes implicated in cancer promotion (based on a survey of published literature), but not previously studied in RMS (Fig. 3B).

**Fig. 4:**
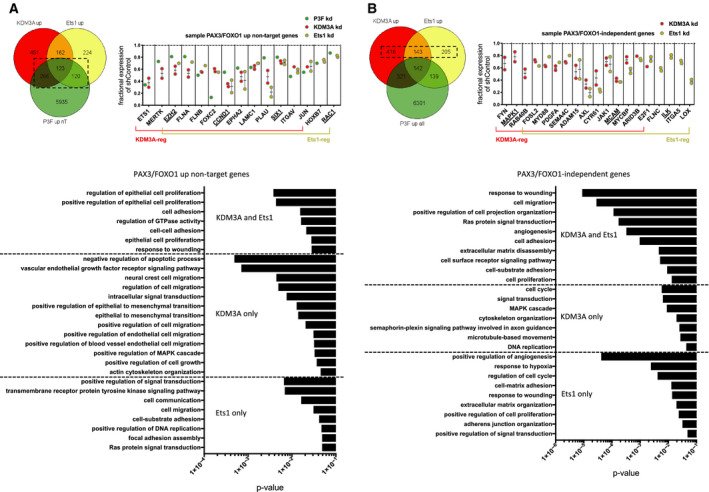
KDM3A and Ets1 additionally regulate known and candidate disease‐promoting genes not under direct control of P3F. (A) Overlap (Venn) analysis of KDM3A and Ets1 ‘up’ transcriptomes in both Rh30 and Rh41 cells (as inferred from genes down in expression upon KDM3A or/and Ets1 KD, in both cell lines), and PAX3/FOXO1 indirectly activated genes, as inferred from genes down upon PAX3/FOXO1 KD, but not identified as PAX3/FOXO1 direct targets by Gryder *et al*.[7] (‘P3F up nT’; dashed box: gene overlaps of interest). Left: Venn diagram data. Right: fractional expression data for selected known (underlined) and novel candidate (all others) disease‐promoting genes in FP‐RMS. For KDM3A and Ets1, each data point represents mean fractional expression (KDM3A‐sh1 and KDM3Ash‐2, or Ets1‐sh1 and Ets1‐sh2, relative to shControl, each *n* = 3) in one cell line; mean and standard error of the mean of fractional expression in both cell lines is also shown; data from effects of PAX3/FOXO1 KD on gene expression are from Gryder *et al*. [7]. Bottom panel: GO analysis of overlapping gene groups, as determined using DAVID. (B) Same overlap (Venn) and GO analyses as ‘A’, but focused on genes not dependent on PAX3/FOXO1 (not decreased in expression upon PAX3/FOXO1 KD) [7] (dashed box: gene overlaps of interest); sample KDM3A and Ets1 fractional gene expression data are plotted as in ‘A’.

PAX3/FOXO1 depletion studies reveal numerous additional genes that are dependent on PAX3/FOXO1 for expression, but are not identified as direct PAX3/FOXO1 targets [7], and may thus represent genes activated by the oncofusion through indirect mechanisms. Gene overlap analysis of this group with our transcriptome data shows that a subset (~ 10%) of such genes are also under positive regulatory control of KDM3A or/and Ets1 in FP‐RMS (Fig. 4A). GO analysis of this coregulated group reveals representation of biological pathways related to cancer progression, including cell growth, survival, and motile properties (Fig. 4A). Included in this group are both genes previously implicated in RMS pathogenesis (EZH2, CCND1, SIX1, and RAC1) [1, 24, 32], and numerous additional cancer‐promoting genes (based on survey of published literature) not previously studied in RMS (a sample of this gene group is shown in Fig. 4A). The KDM3A and Ets1 upregulated transcriptomes, alone and together, also contain numerous additional genes implicated in cancer promotion, which are not dependent on PAX3/FOXO1 for expression (Fig. 4B). GO analysis of this group shows representation of biological pathways related to cell proliferation and motility, and genes that have been previously implicated in FP‐RMS pathogenesis (MAPK1, ILK, MCAM) [33, 34] [12], as well as numerous other cancer‐promoting genes (based on literature survey). This suggests that the disease‐promoting effects of KDM3A and Ets1 in FP‐RMS also involve PAX3/FOXO1‐independent mechanisms.

Taken together, our transcriptome analyses indicate that KDM3A and Ets1 contribute to disease‐promoting gene expression in FP‐RMS both as coregulators of P3F‐dependent gene expression, and through P3F‐independent mechanisms.

### 3.5 The PAX3/FOXO1, KDM3A, Ets1 coregulated, and superenhancer‐associated gene MEST is a disease‐promoting factor in FP‐RMS

**Fig. 5:**
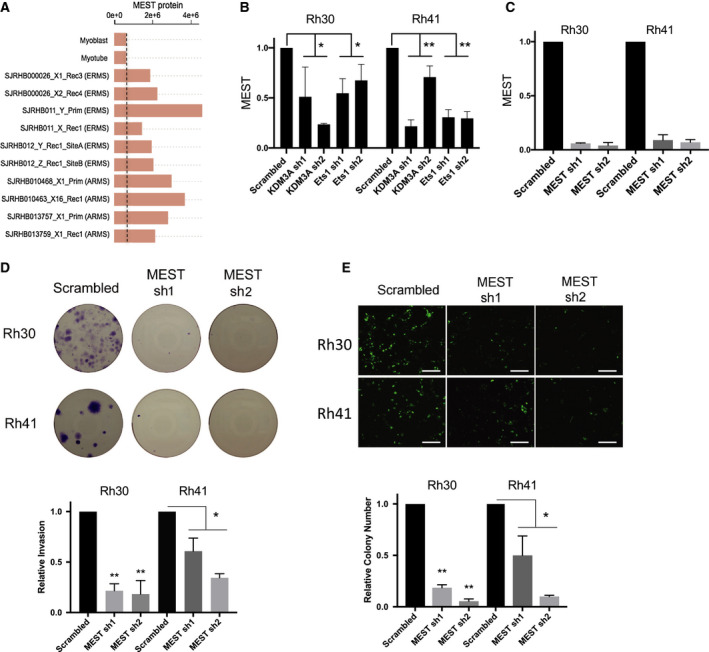
MEST is a disease‐promoting factor in FP‐RMS. (A) MEST expression in RMS patient tumors (from St Jude Children's Research Hospital Pediatric Cancer (PeCan) database (https://pecan.stjude.cloud)). (B) Validation of MEST regulation by KDM3A and Ets1 in Rh30 and Rh41 cells (qRT‐PCR; mean and standard deviation (*n* = 3); *P < 0.05, **P < 0.01 (two‐way student *t*‐test with unequal variance). (C) MEST shRNA‐mediated depletion in Rh41 and Rh30 cells [qRT‐PCR; mean and standard deviation (*n* = 3)]. Effects of MEST depletion on (D) clonogenic growth and (E) transendothelial invasion, performed and analyzed as in Figure 1 (mean and standard error of the mean (*n* = 3); *P < 0.05, **P < 0.01 [1‐way ANOVA with multiple comparisons (Rh30); two‐way student *t*‐test with unequal variance (Rh41)]; scale bars in ‘E’ = 50 µm).

In recent PAX3/FOXO1 cistrome characterization studies [7], the gene MEST (mesoderm‐specific transcript; PEG1) was found to be associated with one of the highest ranked SEs. MEST is an imprinted, developmentally expressed gene of poorly understood function [35]. MEST expression is upregulated in both subtypes of RMS ([30] and Fig. 5A), and, in FP‐RMS, is under direct positive regulatory control of PAX3/FOXO1 ([7] and Fig. 3A). We verified regulation of MEST expression by KDM3A and Ets1 in Rh30 and Rh41 cells (Fig. 5B). To evaluate potential functional roles of MEST in FP‐RMS, we examined the effects of its shRNA‐mediated depletion in Rh30 and Rh41 cells (Fig. 5C). Phenotypic studies revealed that MEST depletion results in potent inhibition of colony formation (Fig. 5D) and transendothelial invasion (Fig. 5E), in both FP‐RMS cell lines. These findings support a disease‐promoting role of MEST in FP‐RMS.

### 3.6 KDM3A and Ets1 impact activation of MEST promoter and PAX3/FOXO1‐associated superenhancer elements

**Fig. 6:**
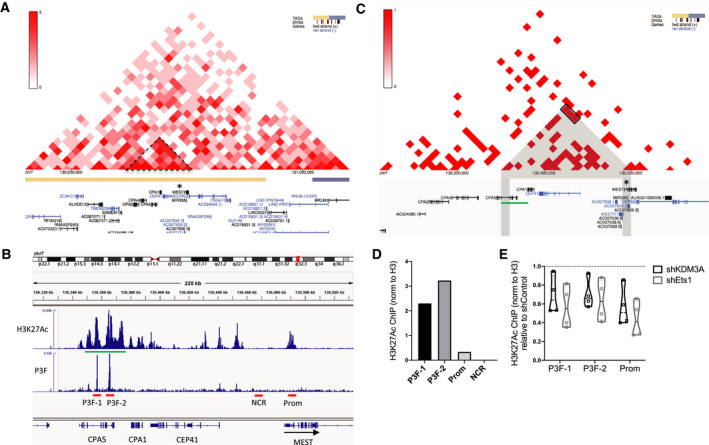
KDM3A and Ets1 contribute to regulatory control of P3F‐bound MEST SE in FP‐RMS. (A) Chromosome Conformation Capture data from Rh30 FP‐RMS cells (Hi‐C data from ENCODE3, generated by Dekker Laboratory, and visualized in http://promoter.bx.psu.edu/hi‐c/view.php, at 40 kbp resolution; heat map scale bar, for strength of corresponding regional interactions, on left); dashed triangle highlights region of strong interactions within TAD (yellow bar below) containing MEST gene (asterisk). (B) Cistrome data at the MEST genomic locus (from [7] via CistromeDB (http://cistrome.org/db), visualized in the Integrated Genomics Viewer (IGV); region shown ~ 200 kb (kilobases; genomic coordinates at top)). Green bar denotes PAX3/FOXO1 (P3F) associated SE region, as defined in [7]. Red bars denote loci interrogated by ChIP studies in ‘D’ and ‘E’, including PAX3/FOXO1‐associated loci (P3F‐1 and −2), MEST promoter region (Prom) and a negative control region. (C) Same data as in ‘A’, visualized at 10 kbp resolution; gray mask denotes interactions between PAX3/FOXO1‐associated SE region (green bar) and MEST promoter region (asterisk), from ‘B’; corresponding interaction heat map data are highlighted by black rectangle. (D) Representative H3K27Ac ChIP signal for the regions indicated in ‘B’ (% input H3K27Ac/ % input total H3; control Rh30 cells; *n* = 2, representative data shown). (E) H3K27Ac levels in shKDM3A and shEts1 cells, relative to shControl cells (violin plot of four independent ChIP experiments (*n* = 4), plotted as % input H3K27Ac/ % input total H3; Rh30 cells).

Chromosome Conformation Capture studies in Rh30 cells (Hi‐C data from ENCODE3, generated by Dekker Laboratory, and visualized in http://promoter.bx.psu.edu/hi‐c/view) show that the MEST genomic locus is part of a Topological Association Domain (TAD) in FP‐RMS (Fig. 6A, yellow bar). Within this TAD, MEST is located in a ~ 200 kbp region showing strong and extensive interactions by 3C (Fig. 6A, dotted triangle), and clusters of enhancer elements showing strong activation (high levels of H3K27 acetylation; Fig. 6B, green bar). These regulatory element clusters comprise one of the top‐ranked SEs in FP‐RMS [7]. Two of the most highly active regulatory elements within this SE show strong binding of PAX3/FOXO1 (Fig. 6B [7]). Rh30 Hi‐C data, visualized at higher resolution (Fig. 6C), show contacts (gray mask and black box) between this PAX3/FOXO1‐bound SE region (green bar) and the MEST promoter region (asterisk). Our chromatin immunoprecipitation (ChIP) analyses show that KDM3A and Ets1 depletion each results in decreased H3K27 acetylation at both PAX3/FOXO1‐bound regulatory elements, as well as the MEST promoter region (Fig. 6D,E). This suggests that KDM3A and Ets1 may regulate MEST expression through promoter and PAX3/FOXO1‐associated SE mechanisms.

## 4 Discussion

Fusion‐positive RMS (FP‐RMS) is an aggressive disease. Like ES [36, 37, 38], another aggressive, fusion oncogene‐driven, cancer of mesenchymal origin affecting children and young adults, FP‐RMS has recently emerged as having a strong epigenetic basis [7]. However, similar to ES, a great deal remains to be learned about critical epigenetic mechanisms, and key downstream disease‐promoting pathways, contributing to aggressive biology in FP‐RMS [39].

Our previous studies identified the epigenetic regulator KDM3A as a potent novel disease‐promoting factor in ES and RMS, and the Ets1 transcription factor as a downstream mechanism contributing to KDM3A effects [12, 14, 40]. In the present study, we show that, in FP‐RMS, Ets1 itself is an important promoter of disease‐relevant phenotypes, namely growth, invasion, and metastasis. In keeping with the similarities in their phenotypic effects, and epistatic relationship, we show that KDM3A and Ets1 control overlapping groups of genes in FP‐RMS. Strikingly, we find that both transcriptomes also strongly overlap with that of the PAX3/FOXO1 driver oncofusion.

Recent characterization of the FP‐RMS cistrome showed that PAX3/FOXO1 acts predominantly as a transcriptional activator, exerts such action mainly through distal enhancer elements, including SEs, and utilizes myogenic transcription factor networks to enforce its effects [7]. Our findings that Ets1 expression is under positive regulatory control of P3F, and contributes to the PAX3/FOXO1‐controlled transcriptome, identify Ets1 as a novel transcription factor enforcing the disease‐driving effects of P3F in FP‐RMS. Ets1 is a known promoter of invasive and metastatic phenotypes in other cancers [19]. It is notable that many of the genes activated by Ets1 in FP‐RMS, both PAX3/FOXO1‐dependent and independent, have roles in metastasis promotion. These findings, coupled with Ets1 phenotypic effects demonstrated herein, suggest that Ets1 may be a critical player in FP‐RMS aggressive disease biology. Interestingly, in ES, Ets1 is repressed by the EWS/Fli1 oncofusion, which also inhibits ES invasive and metastatic properties [41, 42]. The manner in which Ets1 regulation by PAX3/FOXO1 and EWS/Fli1 tracks with the effects of the respective oncofusions on metastatic phenotypes suggests that Ets1 may have a more general, important role in sarcoma metastasis.

Our transcriptome analyses reveal that KDM3A and Ets1 also control the expression of many genes that have known or presumptive roles in FP‐RMS promotion, but are not dependent on PAX3/FOXO1 for expression. As in the case of PAX3/FOXO1‐dependent genes above, many such PAX3/FOXO1‐independent genes are implicated in metastasis. The KDM3A/Ets1 axis thus exerts effects that not only reinforce the action of PAX3/FOXO1, but also complement PAX3/FOXO1 effects, in FP‐RMS promotion and progression. This in turn suggests that KDM3A/Ets1 axis targeting could be an effective means to inhibit aggressive FP‐RMS phenotypes *and* could couple favorably with other approaches aimed at inhibiting PAX3/FOXO1 action. PAX3/FOXO1‐driven FP‐RMS pathogenesis also involves inhibitory effects on myogenic differentiation. This is less well understood mechanistically and may entail both transcriptional repressive mechanisms, as well as indirect effects of cells transitioning to a more proliferative state. KDM3A and Ets1 depletion each also results in increased expression of PAX3/FOXO1‐repressed myogenic genes (Figs 2C and S2). This represents another potential mechanism by which KDM3A/Ets1 axis targeting could be used to inhibit PAX3/FOXO1 effects.

KDM3A and Ets1 phenotypes and transcriptomes, defined in this and our previous studies [12], provide a new perspective for understanding and dissecting disease‐promoting gene expression in FP‐RMS. As an initial insight into such mechanisms, our H3K27Ac ChIP studies suggest that KDM3A and Ets1 may control the activation of the disease‐promoting gene MEST through both promoter and PAX3/FOXO1‐associated SE regulatory elements. Possible mechanisms, which will be interesting and important to delineate in future studies, could include recruitment of P300 or other factors with acetyltransferase activity to these elements, as observed in other contexts [43, 44]. MEST is also upregulated by loss of imprinting (LOI) in RMS [30], which likely additionally contributes to its increased expression in FP‐RMS. Upregulation of MEST expression via LOI is also observed in other cancers [45, 46, 47]. Developmentally, MEST plays important roles in embryonic growth, and neuronal migration and development [35, 48, 49], and our studies show that MEST exerts both pro‐growth and pro‐invasive effects in FP‐RMS. The mechanisms of action of MEST in RMS remain to be defined. One interesting possibility for MEST growth‐promoting effects could be inhibition of Wnt signaling. The Wnt pathway has been shown to inhibit growth, and self‐renewal, properties in FN‐RMS [50], while the Wnt inhibitor SFRP3 has been demonstrated to promote FP‐RMS growth [51]. MEST has been shown to inhibit Wnt signaling during adipocytic differentiation [52]. Thus, downregulation of growth‐inhibitory Wnt activity could be one mechanism for MEST growth‐promoting effects in RMS. Given the high expression of MEST, and inhibitory role of Wnt signaling, in both RMS subtypes, such a mechanism could play an important role in FP‐RMS as well as FN‐RMS.

## 5 Conclusion

In summary, we show that the KDM3A/Ets1 epigenetic axis importantly contributes to disease‐promoting gene expression and phenotypes in FP‐RMS, including PAX3/FOXO1‐dependent and PAX3/FOXO1‐independent genes with roles in metastasis and disease progression. Further understanding of this axis, and development of ways to inhibit its action, could provide a new approach to targeting the aggressive properties of FP‐RMS.

## Conflicts of interest

The authors have no conflicts of interest to disclose.

## Author contributions

LMS and PJ conceived and designed the study. LMS, TSM, JCB, and PJ developed the methodology. LMS, HMH, JKP, and JH acquired the data. LMS, HMH, JKP, AG, JCC, and PJ analyzed and interpreted the data. LMS, HMH, JKP, TSM, JH, AG, JCC, JCB, and PJ wrote, reviewed, and/or revised the manuscript. PJ supervised the study.

## Supporting information


**Fig. S1**. KDM3A and Ets1 transcriptome overlaps in Rh30 and Rh41 cells.

**Fig. S2**. Myogenic genes in KDM3A/Ets1 ‘down’ transcriptomes.

**Table. S1**. PCR primers.

**Table. S2**. KDM3A and Ets1 transcriptome data.

## Data Availability

Gene expression profiling data have been deposited into the NCBI Gene Expression Omnibus database (accession number GSE153852).
