# Supplementary material for: KDM3A/Ets1 epigenetic axis contributes to PAX3/FOXO1‐driven and independent disease‐promoting gene expression in fusion‐positive Rhabdomyosarcoma
Source: Mol Oncol. 2020 Aug 5;14(10):2471–86. doi: 10.1002/1878-0261.12769 (PMC7530783; doi:10.1002/1878-0261.12769)
Supplement: Supplementary file 1 — Fig. S1. KDM3A and Ets1 transcriptome overlaps in Rh30 and Rh41 cells. [file MOL2-14-2471-s001.pdf]

positively regulated genes

| | KDM3A up | | | Ets1 up | | |
|---|---|---|---|---|---|---|
| | # | total | % | # | total | % |
| **Rh41 and Rh30 (relative to Rh41)** | 1022 | 2798 | 36.53% | 629 | 1910 | 32.93% |
| **Rh41 and Rh30 (relative to Rh30)** | 1022 | 1640 | 62.32% | 629 | 1177 | 53.44% |

| **Rh41** | | **KDM3A up** | **Ets1 up** |
|---|---|---|---|
| | **#** | 2798 | 1910 |
| **KDM3A and Ets1** | 1111 | 39.71% | 58.17% |
| **KDM3A only** | 1687 | 60.29% | |
| **Ets1 only** | 800 | | 41.88% |

| **Rh30** | | **KDM3A up** | **Ets1 up** |
|---|---|---|---|
| | **#** | 1640 | 1177 |
| **KDM3A and Ets1** | 612 | 37.32% | 52.00% |
| **KDM3A only** | 1028 | 62.68% | |
| **Ets1 only** | 565 | | 48.00% |

| **Rh41 and Rh30** | | **KDM3A up** | **Ets1 up** |
|---|---|---|---|
| | **#** | 1022 | 629 |
| **KDM3A and Ets1** | 285 | 27.89% | 45.31% |
| **KDM3A only** | 455 | 44.52% | |
| **Ets1 only** | 197 | | 31.32% |

negatively regulated genes

| | KDM3A down | | | Ets1 down | | |
|---|---|---|---|---|---|---|
| | # | total | % | # | total | % |
| **Rh41 and Rh30 (relative to Rh41)** | 814 | 2178 | 37.37% | 552 | 1804 | 30.60% |
| **Rh41 and Rh30 (relative to Rh30)** | 814 | 1664 | 48.92% | 552 | 1226 | 45.02% |

| **Rh41** | | **KDM3A down** | **Ets1 down** |
|---|---|---|---|
| | **#** | 2178 | 1804 |
| **KDM3A and Ets1** | 880 | 40.40% | 48.78% |
| **KDM3A only** | 1298 | 59.60% | |
| **Ets1 only** | 924 | | 51.22% |

| **Rh30** | | **KDM3A down** | **Ets1 down** |
|---|---|---|---|
| | **#** | 1664 | 1226 |
| **KDM3A and Ets1** | 615 | 36.96% | 50.16% |
| **KDM3A only** | 1048 | 62.98% | |
| **Ets1 only** | 611 | | 49.84% |

| **Rh41 and Rh30** | | **KDM3A down** | **Ets1 down** |
|---|---|---|---|
| | **#** | 814 | 552 |
| **KDM3A and Ets1** | 254 | 31.20% | 46.01% |
| **KDM3A only** | 359 | 44.10% | |
| **Ets1 only** | 172 | | 31.16% |

**Supplemental Figure S1. KDM3A and Ets1 transcriptome overlaps in Rh30 and Rh41 cells.** Gene overlap analysis of KDM3A and Ets1-regulated transcriptomes in Rh30 and Rh41 FP-RMS cells (positively and negatively regulated genes are inferred from genes down and up, respectively, upon KDM3A and Ets1 knockdown, as in Figure 2A).
