# Supplementary material for: KDM3A/Ets1 epigenetic axis contributes to PAX3/FOXO1‐driven and independent disease‐promoting gene expression in fusion‐positive Rhabdomyosarcoma
Source: Mol Oncol. 2020 Aug 5;14(10):2471–86. doi: 10.1002/1878-0261.12769 (PMC7530783; doi:10.1002/1878-0261.12769)
Supplement: Supplementary file 2 — Fig. S2. Myogenic genes in KDM3A/Ets1 ‘down’ transcriptomes. [file MOL2-14-2471-s002.pdf]

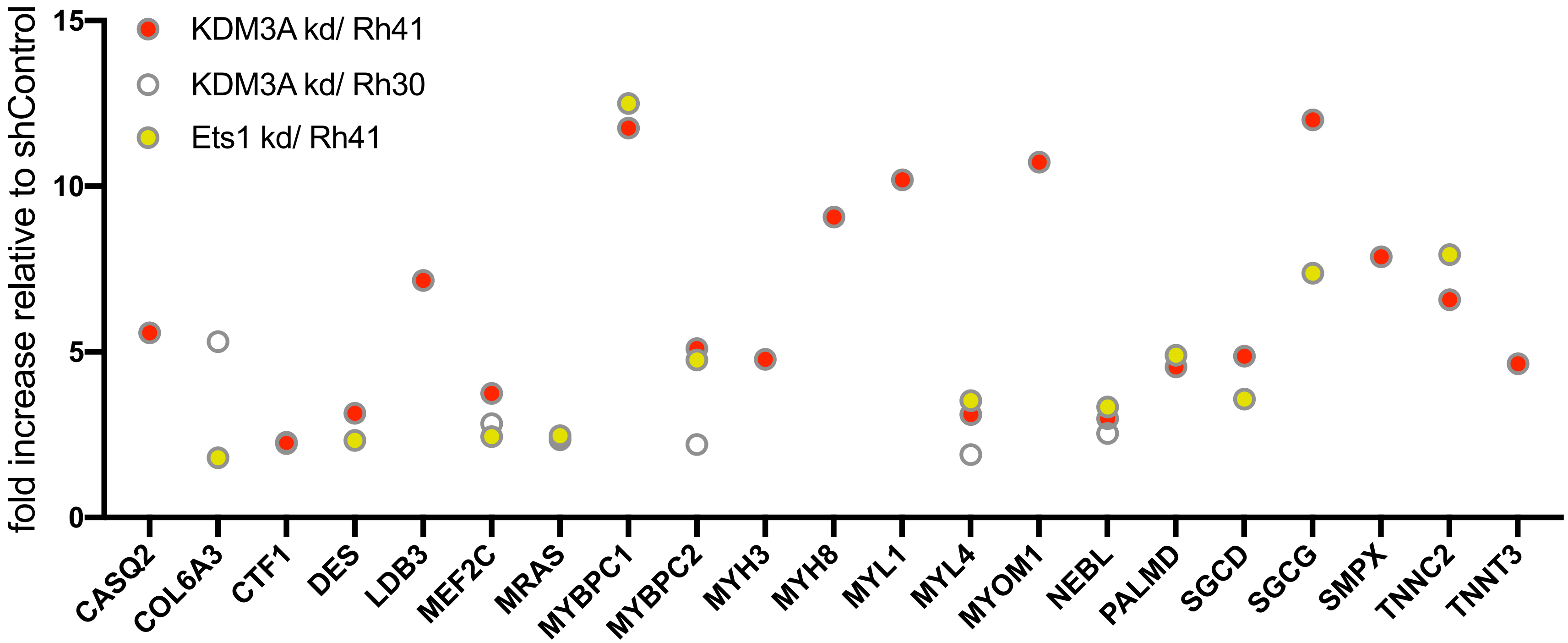

Supplemental Figure S2. Myogenic genes in KDM3A/Ets1 “down” transcriptomes. Genes in KDM3A and Ets1 “down” transcriptomes (as inferred from genes up with KDM3A/Ets1 knockdown) also present in the core enrichment (leading edge) of GSEA data in Figure 2C (Ebauer Myogenic Targets of PAX3/FOXO1 Fusion [53]). Data are plotted similarly to Figures 3 and 4 (each data point representing fold-increase in mean expression [KDM3A-sh1 and KDM3Ash-2, or Ets1-sh1 and Ets1-sh2, relative to shControl, each n=3] in the indicated cell line).
